# Metformin-associated lactic acidosis treated successfully by peritoneal dialysis in a resource limited setting: case report

**DOI:** 10.11604/pamj.2019.32.112.18271

**Published:** 2019-03-11

**Authors:** Khaled Elmezughi, Chukwuma Ekpebegh

**Affiliations:** 1 Department of Internal Medicine, Walter Sisulu University, Mthatha, South Africa

**Keywords:** Lactic acidosis, metformin overdose, metformin toxicity, peritoneal dialysis, South Africa

## Abstract

Metformin is a commonly used treatment modality in type 2 diabetes mellitus with lactic acidosis as a rare but life-threatening side effect. In this case report we highlight the importance of recognizing this uncommon side effect and the treatment options in a resource limited situation. We present a 14-year-old African girl who ingested an unknown amount of metformin intentionally after an argument with her mother. She was referred late to our institution in severe lactic acidosis. Lactic acidosis resolved with appropriate treatment including peritoneal dialysis. We conclude that in resource constrained settings, peritoneal dialysis may be used for metformin associated lactic acidosis with favourable outcome.

## Introduction

Metformin is the recommended first-line therapy for patients with type 2 diabetes mellitus especially those who are overweight [1]. Despite its being generally safe, metformin may lead to a rare but life-threatening adverse reaction: metformin-associated lactic acidosis (MALA) [2]. MALA is defined as the presence of pH < 7.35, blood lactate >2.0 mmol/L and PaCO2 < 42 mmHg within the context of recent metformin exposure [3-5]. It is associated with a mortality rate of up to 50% [3, 6, 7]. MALA is a rare condition with an estimated incidence of 2-9 patients per 100,000 patients receiving metformin per year [3, 7]. In the largest case series published in the literature to date, the authors reported no more than dozens of cases over years of observation [8]. The etiology of lactic acidosis is multifactorial and uncertain: inhibition of gluconeogenesis by reducing hepatic lactate uptake, inhibition of pyruvate dehydrogenase activity and mitochondrial-reducing agent transport leading to increased metabolism of pyruvate into lactate [3, 9]. It also impairs lactate clearance by the liver through the inhibition of complex 1 of the mitochondrial respiratory chain [5]. MALA usually occurs with impaired renal function but may occur with preserved renal function in the setting of massive metformin ingestion [3, 4]. Voluntary poisoning is rare, and its characteristics and prognosis are seldom reported in the literature [5]. Intermittent haemodialysis is the most frequently reported treatment modality and early continuous renal replacement therapy (CRRT) has been suggested as an alternative along with supportive measures [3]. Although there is limited evidence regarding slow CRRT, it is preferred in patients with haemodynamic instability as it is better tolerated than haemodialysis [10]. Peritoneal dialysis (PD) has been used in the past as a treatment modality for acute intoxications as it is used in the settings of acute kidney injury (AKI) or end-stage renal disease (ESRD) [11], however, to our knowledge no reports have been described in using it for metformin overdose. We describe a case of MALA in a young female, who intentionally ingested an unquantifiable amount of metformin in a suicidal attempt. Although there was profound metabolic acidosis, she fully recovered with fluid resuscitation, bicarbonate administration and rapid institution of peritoneal dialysis.

## Patient and observation

A 14-year old girl was referred to Nelson Mandela Academic Hospital (NMAH) from a district level Hospital few hours after intentional ingestion of a large unquantifiable amount of metformin tablets. This was following an argument with her mother who is diabetic and on metformin treatment. The patient herself was not known with diabetes or psychiatric illness and had no significant past medical history. She was not on any chronic medications. The notable findings on physical examination were acidotic breathing, sinus tachycardia of 120 beats per minute, hypotension (BP 80/45 mmHg) and abdominal tenderness. She was drowsy but arousable. Arterial blood gas on arrival to NMAH showed PH = 7.05, Po2 =115 mmHg= PCo2 = 27.5 mmHg, Base Excess = -23 mmol/L, bicarbonate = 7.6 mmol/, potassium = 6.7 mmol/L, capillary glucose reported low on the glucometer. Lactate level was rejected by the laboratory because of it was not sent on ice. Her other laboratory results at presentation were marked leukocytosis, severe metabolic acidosis, hyperkalemia, hyponatremia as well as mild elevation of the liver enzymes as depicted in **Table 1**. Electrocardiogram showed peaked T-waves and sinus tachycardia. Chest radiograph was unremarkable. Blood cultures and toxicology screen for paracetamol and salicylate levels were negative. A diagnosis of metformin associated lactic acidosis was made and 100 grams of activated charcoal was administered in the emergency department. She also received 20mls boluses of 50% dextrose several times and was subsequently maintained on intravenous 5% dextrose infusion. Her renal function started deteriorating on the second day after admission in keeping with AKI; (urea 12.6 mmo/L, Creatinine = 251 umol/L, K=7.2 mmol/L) most likely due to metformin toxicity. Hyperkalemia was initially treated with the combination of intravenous calcium gluconate and insulin-glucose to facilitate intracellular shift of potassium. She also received 100mls of 8.4% sodium bicarbonate via a central line. Due to lack of bed in ICU/high care, it was decided that the patient be treated in our acute peritoneal dialysis unit. Peritoneal dialysis was started because of limited availability of haemodialysis. After 45 cycles of peritoneal dialysis in 5 days, she achieved recovery of renal function with restoration of urine output, acid-base balance and complete resolution of lactic acidosis. She was discharged home on the 7^th^ day after admission in very good condition.

**Table 1:** Laboratory results at presentation

	Patient’s value	Normal range
Hb (g/L)	15.1	12-15
WBC [x10^9^ /L]	36.53	3.9-12.6
Platelets [x10^9^ /L]	402	186-454
HCO3 − [Venous] [mmol/L]	2	23-29
Na+ [mmol/L]	133	136-145
K+ [mmol/L]	6.9	3.5-5.1
Cl− [mmol/L]	84	98-107
Anion Gap [mmol/L]	54	9-16
Urea [mmol/L]	4.3	1.4-5.4
Creatinine [umol/L]	71	40-72
Total protein [g/L]	86	57-80
Albumin [g/L]	49	29-42
Bilirubin [umol/L]	8	5-21
ALT [U/L]	38	5-20
GGT [U/L]	35	4-24
Lactate [mmol/L]	Rejected (not sent on ice)	0.5-2.2

ALT, alanine aminotransferase; AST, aspartate aminotransferase; GGT, gamma-glutamyl transferase

## Discussion

Metformin, a biguanide, was first used in the 1950s in Europe and Canada and, since 1995, in the United States. It is the most widely used oral antihyperglycemic agent in the world and is the first-line medication for the treatment of type 2 diabetes [9]. Metformin is a small 165 Da molecule with an oral availability of 55% and a distribution volume of 1-5 L/kg. The elimination half-life is 8-20 hours in individuals with normal renal function [5]. Despite the increasing use of metformin, the prevalence of MALA is uncommon; 0.03 cases per 1000 patient-years [2]. Suicide with metformin is rare. Intake of 35 g of metformin has been shown to be lethal [12], however, survival following an overdose of 63 g has been reported [13]. In cases of MALA, the serum lactate level does not correlate with prognosis, even with lactate levels as high as 35.5 mmol/L [12]. Our patient developed profound lactic acidosis that was associated with acute kidney injury, severe hyperkalemia and hypoglycemia. The high anion gap metabolic acidosis is likely due to both lactic and ureamic acidosis. Although serum lactate level was rejected by the laboratory at presentation, subsequent level was found to be still elevated (3.3 mmol/L); normal ranges between 0.5-2.2 mmol/L. Serum metformin assay is not available at our institution, however, other causes of elevated serum lactate including sepsis and tissue ischemia were excluded and there was no evidence of hepatic disease or other toxin ingestions. Our patient presented with classical symptoms of MALA within the context of metformin overdose. Although renal failure is a risk factor for MALA. Our 14-year-old patient's renal function was normal initially, however, it started deteriorating on the second day after presentation and is likely to be caused by metformin overdose. Transient hypotension in our patient responded promptly to intravenous fluids. The pathophysiologic mechanisms of hypotension in MALA include negative inotropic effects and increased systemic vascular resistance with acidosis. Profound hypotension that is unresponsive to fluid therapy may require vasopressors [5]. The use of sodium bicarbonate is controversial and not fully validated in the clinical scenario of lactic acidosis and haemodynamic instability, regardless of the aetiology [5]. Prompt treatment of acidosis in our patient with the combination of fluids, bicarbonate and peritoneal dialysis likely prevented the need for vasopressors. Metformin overdose can be treated easily with standard or high-flux haemodialysis to correct the lactic acidosis as well as to remove the medication [14]. Although mortality rate is high in cases of MALA [3, 8], our patient had a favorable clinical outcome likely from the clearance of metformin and lactate during peritoneal dialysis.

## Conclusion

In resource constrained settings where haemodialysis is not readily available, peritoneal dialysis may be used for the treatment of metformin associated lactic acidosis with a favourable outcome.
